# Immunohistochemical detection of multidrug resistance associated P-glycoprotein in tumour and stromal cells of human cancers.

**DOI:** 10.1038/bjc.1990.256

**Published:** 1990-08

**Authors:** D. Schlaifer, G. Laurent, S. Chittal, T. Tsuruo, S. Soues, C. Muller, J. Y. Charcosset, C. Alard, P. Brousset, C. Mazerrolles

**Affiliations:** Service d'Hématologie, Chu Purpan, Toulouse, France.

## Abstract

**Images:**


*Br. J. Cancer* (1990), **62**, 177–182 © Macmillan Press Ltd., 1990

### Immunohistochemical detection of multidrug resistance associated P-glycoprotein in tumour and stromal cells of human cancers

D. Schlaifer[1], G. Laurent[1], S. Chittal[2], T. Tsuruo[3], S. Soues[4], C. Muller[4], J.Y. Charcosset[4], C. Alard[2], P. Brousset[2], C. Mazerrolles[2] & G. Delsol[2]

[1]*Service d'Hématologie, Chu Purpan, 31059 Toulouse, France;* [2]*Service d'Anatomie-Pathologique et Groupe d'Etude des Lymphomes Malins, Chu Purpan, 31059 Toulouse, France;* [3]*Cancer Chemotherapy Centre, Japanese Foundation for Cancer Research, Kita-ikebukuro, Tokyo 170, Japan; and* [4]*Laboratoire de Pharmacologie et Toxicologie Fondamentales, CNRS, 205 route de Narbonne, 31400 Toulouse, France.*

**Summary** The distribution of Gp 170, a multidrug resistance (MDR) associated glycoprotein, also called P-glycoprotein (P-gp), was examined by immunohistochemistry, using C219 and MRK16 monoclonal antibodies. Sixty-five tumour tissues were studied which included 40 non-lymphoid tumours, 15 chemoresistant non-Hodgkin's lymphomas and 10 Hodgkin's disease. The study was performed on both cryostat and special fixation processed and paraplast embedded (ModAMeX) sections. The latter method preserves fixation-sensitive antigens such as P-gp and allows a more precise morphological identification of neoplastic and non-neoplastic cell populations in contrast to cryostat sections. Immunohistochemical expression of P-gp was expected and confirmed in many non-lymphoid tumours, but stromal macrophages and endothelial cells were also frequently stained in these cases. In non-Hodgkin's lymphomas, cells that were stained with both C219 and MRK16 monoclonal antibodies on cryostat sections were identified as macrophages and endothelial cells and not neoplastic lymphoid cells, by the ModAMeX technique. These findings suggest that the quantitative assessment of MDR RNA by Northern blotting performed on fresh homogenates overestimates the MDR content of neoplastic cells in a number of lymphoid and non-lymphoid tumours. In addition, the mechanism of chemoresistance in non-Hodgkin's lymphomas is less likely to be associated with P-gp expression.

One of the critical limitations in cancer chemotherapy is the presence of either intrinsic, or acquired, drug-resistant population of tumour cells. Multidrug resistance (MDR), is most frequently characterised *in vitro* by an increase of drug efflux and a decrease of drug accumulation in resistant cells, compared to their drug-sensitive parental cells. Concomitant amplification of the human MDR 1 gene, and hence the over expression of a 170 KDa surface membrane glycoprotein, Gp 170, also called P-glycoprotein (P-gp), is usually correlated with MDR (Bradley *et al.*, 1988; Kaye, 1988).

P-gp expresion has been evaluated in many tumour samples, either by using quantitation of MDR 1 RNA by hybridisation probes (Fojo *et al.*, 1987a, 1987b; Goldstein *et al.*, 1989; Moscow *et al.*, 1989) or by the measurement of P-gp over expression by immunoblot using monoclonal antibodies (MoAb) (Bell *et al.*, 1985; Gerlach *et al.*, 1987). P-gp expression has been found to be elevated in untreated intrinsically drug-resistant tumours, such as colon or renal carcinomas and in some recurrent but originally chemosensitive cancers that include non-Hodgkin's lymphomas (Goldstein *et al.*, 1989; Moscow *et al.*, 1989).

However, the cellular heterogeneity of P-gp expression as recently identified in experimental models (Shen *et al.*, 1988), is not clearly apparent by molecular techniques performed with the bulk tumour. Since the presence of resistant cell clusters among a given cell population may influence the clinical outcome of patients, it may be important to use investigational techniques that identify a minority of P-gp expressing cells (Marie *et al.*, 1989). Immunohistochemistry offers the advantages of processing routine specimens and is generally considered to be a reliable, sensitive and rapid method for characterising glycoproteins in the cell membranes of tumour cells (Delsol *et al.*, 1989).

Few immunohistochemical studies have yet emphasised the P-gp expression in human tumour biopsy specimens (Salmon *et al.*, 1989; Dalton *et al.*, 1989; Sugawara *et al.*, 1989). Moreover, these studies have usually been performed with cryostat preparations. In our experience, the precise morphological identification of immunostained cells can be difficult, especially in lymphoid tumours. Several MoAb directed against P-gp have been generated (Kartner *et al.*, 1985; Hamada & Tsuruo, 1986; Scheper *et al.*, 1988). Among these, MRK16 and C219 MoAb were the most extensively used (Thiebaut *et al.*, 1987; Sugawara *et al.*, 1988; Thiebaut *et al.*, 1989). We investigated the reactivities of both C219 and MRK16 MoAb in 65 tumour specimens by using both cryostat and paraplast-embedded preparations. The latter technique combines excellent morphology with preservation of most glycoprotein surface antigens (Delsol *et al.*, 1989). Unexpected expression of P-gp by stromal cells, such as macrophages and endothelial cells was found with this technique. The finding should lead to a reconsideration of the significance of Northern blot for exploring P-gp expression in human cancers.

Correspondence: G. Laurent.
Received 15 December 1989; and in revised form 27 February 1990.

### Materials and methods

### *Tumour specimens and patient selection*

Most tissues were retrieved from our tissue bank with storage temperature of − 80°C. Forty solid tumour samples, which included 5 sarcomas, 23 carcinomas and 12 others, were tested (Table I). The clinical outcome (chemotherapy sensitivity) in these cases was not determined. Ten biopsies of Hodgkin's disease were studied (Table II). The first eight patients were in complete remission after 3 to 12 ABVD or MOPP cycles whereas 2 patients were in progressive disease. Fifteen cases of non-Hodgkin's lymphomas were enrolled in this study (Table III). The patients were selected for their chemoresistance to drug regimens which included both adriamycin and vincristine, known to induce P-gp expression. The lymphomas had been previously phenotyped by standard procedures on cryostat sections by a panel of anti-B anti-T cluster designated monoclonal antibodies (data not shown). In 6 cases (cases 1 to 6), lymph node biopsies were performed for diagnosis. These patients later became fully resistant to 6 to 12 cycles of CHOP-B/M-BACOD front line therapy. In 9 other cases (cases 7 to 15), biopsy specimens had been obtained from patients who were previously treated with 2 to 7 cycles of COP/CHOP-B/M-BACOD and were in relapse at the time of the study, the patients later becoming resistant to salvage therapy.

**Table I** Non-lymphoid tumours (P-gp staining)

| | | *P-gp staining*[a] | | | | | |
|---|---|---|---|---|---|---|---|
| | | *Neo* | | *Mac* | | *End* | |
| *No* | *Histological type* | *C219* | *MRK16* | *C219* | *MRK16* | *C219* | *MRK16* |
| 1 | Unclassified sarcoma | 0 | 0 | 0 | 0 | 0 | 0 |
| 2 | Rhabdomyosarcoma | 0 | 0 | 0 | 0 | 0 | 0 |
| 3 | Rhabdomyosarcoma | 0 | 0 | 0 | 0 | 0 | 0 |
| 4 | Leiomyosarcoma | 0 | 0 | 0 | 0 | 2M | 2M |
| 5 | Leiomyosarcoma | 0 | 0 | 0 | 0 | 0 | 0 |
| 6 | Colon carcinoma | 0 | 0 | 3M | 2M | 0 | 0 |
| 7 | Colon carcinoma | 0 | 0 | 0 | 0 | 0 | 0 |
| 8 | Carcinoma of unknown origin | 1W | 1W | 0 | 0 | 0 | 0 |
| 9 | Carcinoma of unknown origin | 1W | 1W | 0 | 0 | 0 | 0 |
| 10 | Gastric carcinoma | 0 | 0 | 0 | 0 | 0 | 0 |
| 11 | Gastric carcinoma | 0 | 0 | 0 | 0 | 0 | 0 |
| 12 | Gastric carcinoma | 0 | 0 | 0 | 0 | 0 | 0 |
| 13 | Small intestine carcinoma | 1M | 1M | 0 | 0 | 0 | 0 |
| 14 | Renal cell carcinoma | 1M | 1W | 0 | 0 | 0 | 0 |
| 15 | Renal cell carcinoma | 35 | 35 | 0 | 0 | 0 | 0 |
| 16 | Renal cell carcinoma | 2W | 2W | 0 | 0 | 0 | 0 |
| 17 | Ovarian carcinoma | 25 | 25 | 0 | 0 | 0 | 0 |
| 18 | Breast carcinoma | 0 | 0 | 0 | 0 | 0 | 0 |
| 19 | Breast carcinoma | 0 | 0 | 0 | 0 | 0 | 0 |
| 20 | Non-small cell lung carcinoma | 0 | 0 | 2M | 2M | 0 | 0 |
| 21 | Non-small cell lung carcinoma | 0 | 0 | 0 | 0 | 0 | 0 |
| 22 | Non-small cell lung carcinoma | 0 | 0 | 0 | 0 | 0 | 0 |
| 23 | Non-small cell lung carcinoma | 0 | 0 | 0 | 0 | 0 | 0 |
| 24 | Prostate carcinoma | 0 | 0 | 2M | 2M | 0 | 0 |
| 25 | Prostate carcinoma | 0 | 0 | 2M | 2M | 0 | 0 |
| 26 | Prostate carcinoma | 0 | 0 | 2M | 2M | 0 | 0 |
| 27 | Thyroid carcinoma | 0 | 0 | 0 | 0 | 0 | 0 |
| 28 | Liver-cell carcinoma | 2M | 2M | 25 | 25 | 0 | 0 |
| 29 | Astrocytoma | 25 | 25 | 25 | 25 | 0 | 0 |
| 30 | Astrocytoma | 0 | 0 | 35 | 35 | 35 | 2M |
| 31 | Plasmocytoma | 0 | 0 | 2W | 2W | 0 | 0 |
| 32 | Wilm's tumour | 2W | 2W | 0 | 0 | 0 | 0 |
| 33 | Phaeochromocytoma | 0 | 0 | 1M | 25 | 0 | 0 |
| 34 | Phaeochromocytoma | 0 | 0 | 2M | 2M | 0 | 0 |
| 35 | Carcinoid tumour | 0 | 0 | 25 | 25 | 0 | 0 |
| 36 | Melanoma | 0 | 0 | 2W | 2W | 0 | 0 |
| 37 | Melanoma | 0 | 0 | 2W | 2W | 0 | 0 |
| 38 | Melanoma | 0 | 0 | 35 | 25 | 0 | 0 |
| 39 | Skin myelomonocytic tumour | 25 | 25 | 0 | 0 | 0 | 0 |
| 40 | Breast fibroadenoma | 2W | 2W | 0 | 0 | 0 | 0 |

[a]P-gp staining: Neo = neoplastic cells, Mac = macrophages. End = endothelial cells. Number of positive cells: 0 = no positive cell, 1 = $<10\%$ positive cells; 2 = 10–75% positive cells, 3 = $>75\%$ positive cells. Intensity of staining: W = weak, M = moderate, S = strong.

**Table II** Hodgkin's disease, clinical data and results of P-gp staining

| | | | | | | *P-gp staining*[c] | | | | | |
|---|---|---|---|---|---|---|---|---|---|---|---|
| | | | | | | *Neo* | | *Mac* | | *End* | |
| *No* | *Sex-Age* | *Grade* | *Stage* | *Therapy*[a] | *Response*[b] | *C219* | *MRK16* | *C219* | *MRK16* | *C219* | *MRK16* |
| 1 | F-49 | 3 | IVBb | MOPPx6<br>ABVDx6 | CR | 0 | 0 | 35 | 35 | 0 | 0 |
| 2 | M-42 | 1 | IIIAa | MOPPx3<br>ABVDx3 | CR | 0 | 0 | 2W | 2W | 0 | 0 |
| 3 | M-24 | 2 | IIIBb | MOPPx3<br>ABVDx3<br>RT | CR | 0 | 0 | 2W | 2W | 0 | 0 |
| 4 | F-18 | 2 | IIIBb | MOPPx3<br>ABVDx3<br>RT | CR | 0 | 0 | 2W | 2W | 0 | 0 |
| 5 | M-27 | 2 | IIIBb | MOPPx3<br>ABVDx3<br>RT | CR | 0 | 0 | 0 | 0 | 0 | 0 |
| 6 | M-27 | 2 | IIAb | MOPPx3<br>RT | CR | 0 | 0 | 0 | 0 | 0 | 0 |
| 7 | M-61 | 2 | IIIBb | MOPPx3<br>ABVDx3 | CR | 0 | 0 | 0 | 0 | 0 | 0 |
| 8 | F-30 | 2 | IIAa | MOPPx3<br>RT | CR | 0 | 0 | 0 | 0 | 0 | 0 |
| 9 | F-42 | 2 | IVBb | MOPPx6<br>ABVDx6<br>RT | PD | 0 | 0 | 2M | 2M | 1M | 1M |
| 10 | M-62 | Nd | IVBb | MOPPx6<br>ABVDx6 | PD | 0 | 0 | 0 | 0 | 0 | 0 |

Nd = not determined, CR = complete response, PD = progressive disease. [a]MOPP = Mustine, Vincristine, Procarbazine, Prednisone. ABVD = Adriamycin, Bleomycin, Vinblastine, Dacarbazine. RT = Radiotherapy. [b]Response evaluated at the end of the chemotherapy. [c]P-gp staining: key in Table I.

**Table III** Non-Hodgkin's lymphomas, clinical data and results of P-gp staining

| | | | | | P-gp staining | | | | | |
|---|---|---|---|---|---|---|---|---|---|---|
| | | | Therapy[b] | | Neo | | Mac | | End | |
| *No* | *Sex-Age* | *Histology*[a] | *a* | *b* | *C219* | *MRK16* | *C219* | *MRK16* | *C219* | *MRK16* |
| 1 | F-59 | F | no | M-BACODx6 | 0 | 0 | 35 | 35 | 0 | 0 |
| 2 | M-67 | B | no | CHOP-Bx6 | 0 | 0 | 0 | 0 | 0 | 0 |
| 3 | M-69 | G | no | CHOP-Bx4 | 0 | 0 | 2M | 2M | 0 | 0 |
| 4 | F-75 | H | no | CHOP-Bx7 | 0 | 0 | 2M | 2M | 1W | 1W |
| 5 | M-50 | H | no | CHOP-Bx8 | 0 | 0 | 2M | 2W | 0 | 0 |
| 6 | M-66 | E | no | CHOP-Bx4 | 0 | 0 | 0 | 0 | 1W | 1W |
| 7 | M-52 | H | M-BACODx6 | CHOP-Bx6 | 0 | 0 | 0 | 0 | 0 | 0 |
| 8 | F-56 | H | CHOP-Bx6 | M-BACODx1 | 0 | 0 | 2M | 2M | 0 | 0 |
| 9 | M-53 | G | M-BACODx6 | CHOP-Bx4 | 0 | 0 | 25 | 25 | 2W | 2W |
| 10 | M-59 | E | CHOP-Bx6 | M-BACODx6 | 0 | 0 | 1M | 1W | 1M | 1M |
| 11 | F-65 | F | CHOP-Bx6 | M-BACODx9 | 0 | 0 | 1M | 1M | 0 | 0 |
| 12 | F-52 | C | CHOP-Bx7 | M-BACODx2 | 0 | 0 | 35 | 35 | 0 | 0 |
| 13 | M-71 | C | CHOP-Bx6 | M-BACODx4 | 0 | 0 | 0 | 0 | 0 | 0 |
| 14 | F-41 | G | COPx2 | M-BACODx5 | 0 | 0 | 25 | 25 | 0 | 0 |
| 15 | F-55 | A | COPx2 | CHOPx6 | 0 | 0 | 25 | 25 | 0 | 0 |

[a]Histology was given according to the Working Formulation (Anonymous, 1982): A = lymphocytic lymphoma, B = follicular small cleaved cell lymphoma, C = follicular small and large cell lymphoma, E = diffuse small cleaved cell lymphoma, F = diffuse mixed lymphoma, G = diffuse large cell lymphoma, H = immunoblastic lymphoma. [b]Therapy (a) before and (b) after the lymph node biopsy studied here. A or H = adriamycin, B = bleomycin, C = cyclophosphamide, D = dexamethasone, M = methotrexate, D = vincristine, P = prednisone. [c]P-gp staining: key in Table I.

*Non-tumour tissues*

Adrenal, kidney and liver tissue specimens, freshly obtained from necropsies, were investigated for comparison of known P-gp expression. P-gp expression has been reported to occur on normal adrenal cortical and medullary cells, apical portion of proximal tubular cells of the kidney, and canalicular surface of hepatocytes (Thiebaut *et al.*, 1987).

Four cases of benign lymph nodes (3 sarcoidosis and 1 reactive lymphadenitis) and a normal spleen from a case of incidental splenectomy were included in the study.

*Cell lines*

Parental Chinese hamster ovary (CHO) cell line (Aux B1) and the MDR $CH^RC5$ subline (Kartner *et al.*, 1985), kindly provided by Dr R.M. Baker (RPMI, Buffalo, NY), as well as the parental human KB-3-1 and the adriamycin resistant sublines KB-$ADR^R$-5A and KB-A1 (Pastan & Gottesman, 1987), a generous gift of Dr M. Gottesman (NCI. Bethesda, MD), were used as negative and positive controls.

*Monoclonal antibodies*

Two MoAb, C219 (Centocor, Malvern) directed against an internal epitope of P-gp (Kartner *et al.*, 1985) and MRK16, which reacts with the external region of the molecule (Hamada *et al.*, 1986), were used. Two CD68 monoclonal antibodies (Y2/131 from Dr D.Y. Mason, Oxford, UK & KP-1 from Dakopatts, Copenhagen, Denmark) with major cellular reactivity against macrophage-associated antigen (gp110) were used for investigation of the nature of stromal cells. Negative controls were used by the omission of the primary antibody. Simultaneous staining of several different sections with the same antibody served as mutual control.

*Immunohistochemical techniques*

*Tissues preparation* Histological specimens were obtained from the operating room within one hour of resection. Specimens were sliced into three parts. One part was fixed in ethanol based Bouin's fluid (Dubosq-Brazil) and processed for routine histopathology. The second part was snap-frozen in liquid nitrogen and stored at − 80°C until used. The third part was processed according to the ModAMeX method, a modification of the AMeX method (Sato *et al.*, 1986), described recently in detail elsewhere (Delsol *et al.*, 1989). Briefly, tissues for the ModAMeX method were sliced approximately 2–3 mm thick for fixation in cold acetone at 4°C containing protease inhibitors. Fragments were then usually left at − 20°C to fix overnight. Tissues were then dehydrated in acetone-containing protease inhibitors at 4°C for 15 minutes followed by immersion in acetone at room temperature for 15 minutes. Sections were cleared in methyl benzoate for 15 minutes and subsequently in xylene for 15 minutes. Embedding was performed in a low melting point paraplast (X-Tra, Carlo, Erba).

*Immunostaining procedure* A method for cryostat sections has been described previously (Laurent *et al.*, 1986). The ModAMeX preparations were warmed to 54°C for 2 minutes before deparaffinisation in xylene for 10 minutes. Sections were then immersed in acetone for 4 minutes, in either Tris-buffered saline (TBS) plus acetone or phosphate buffered saline (PBS) plus acetone for 2 minutes followed by immersion in TBS or PBS for 4 minutes and finally in TBS or PBS plus bovine albumin (1%) for 4 minutes. Both cryostat and ModAMeX sections were stained by the APAAP method (Cordell *et al.*, 1984). MRK16 and C219 MoAb were used at $4\ \mu g\ ml^{-1}$ final concentrations. All sections were read by one of us (GD) in batches. Even though no quantitation could be applied to the assessment of staining reactivities, the intensity of the reaction product could be semi-objectively classified as weak, moderate or strong which is the notation that has been used in the presentation of results.

*Cell lines* CHO and KB cell lines were suspended in culture medium and centrifuged (14 *g* for 5 minutes) with cytospin 2 (Shandon Inc, Pittsburgh, PA). After fixation with undiluted acetone and chloroform for 15 minutes, the cell lines were processed in the same manner as tissues for cryostat sections as well as by immunoperoxidase (Laurent *et al.*, 1986) and APAAP techniques (Cordell *et al.*, 1984).

### Results

The feasibility of immunohistochemical assessment of the reactivities of MoAb against P-gp was first confirmed with the cell lines. The parental cell lines, CHO Aux B1 and KB-3-1 were negative for both C219 and MRK16 MoAb whereas strong positive reactions were obtained with resistant cell lines, CHO MDR $CH^RC5$ as well as KB-$ADR^R$-5A and KB-A1 (adriamycin-resistant) (data not shown).

Positive staining, denoting P-gp expression by neoplastic cells of non-lymphoid tumours, was obtained in 12 out of 40 cases (30%), namely, in renal-cell carcinoma, liver-cell carcinoma, ovarian cacinoma, astrocytoma, Wilm's tumour, cutaneous localisation of an acute myelomonocytic leukaemia and a breast fibroadenoma (Table I). In the P-gp expressing tumours, malignant cells were diffusely stained with variable, but generally moderate intensity. The pattern of reactivities of neoplastic cells bore no relation to the type of the neoplasm. We were surprised to find exclusive positivity on macrophages, with the two anti-P-gp antibodies, in 13 out of 40 cases. In these cases, where P-gp expression was solely encountered on macrophages, the neoplastic cells were completely negative. The intensities of the reaction product on the macrophages varied between tumour types but were comparable for a given tumour for the two antibodies. In two instances (liver cell carcinoma and astrocytoma), P-gp expression was found on both neoplastic cells and macrophages (Table I). P-gp was expressed exclusively by some endothelial cells only in one instance (leiomyosarcoma) and simultaneously with macrophages in one other case (astrocytoma).

In ten cases of Hodgkin's disease (8 chemosensitive and 2 chemoresistant), Reed-Sternberg cells were unstained in all cases while macrophages and endothelial cells were reactive with both C219 and MRK16 MoAb in five cases. The number of cases in each histological type are too few to draw meaningful correlation with histopathological subtypes.

In non-Hodgkin's lymphomas, a clear immunostaining, but with a variable proportion of cells in each case, was obtained with both C219 and MRK16 MoAb in 13 out of 15 cases (86%) on cryostat sections. Positive cells were either in small clusters or scattered throughout microscopic fields. The intensity of the immunostaining varied but was often strong. Accurate cytological identification of stained cells remained uncertain on cryostat sections. However, these immunoreactive cells were positively identified as macrophages in ModAMeX prepared sections (Figure 1). In addition to the characteristic morphology, some immunostained cells showed tingible bodies in their cytoplasm. The neoplastic lymphoid cells were clearly negative in all cases of non-Hodgkin's lymphoma (Table III). The reactivity of macrophages by anti-P-gp antibodies was further confirmed by simultaneous staining of serial sections in three cases with CD68 antibodies (Figure 2). It needs to be noted though, that the proportion of macrophages stained with the CD68 was greater than those stained by the two anti-P-gp MoAb.

Five specimens of non-malignant lymphoid tissue (3) sarcoidosis, 1 non-specific reactive lymphadenitis, and 1 normal spleen) were examined. Normal and reactive lymphocytes were negative for P-gp expression both in lymph nodes and in the spleen. Variable and inconsistent staining was observed on endothelial cells of capillaries in lymph node and spleen with the two antibodies. In lymph node, but not in the spleen, numerous epitheloid macrophages, mainly in aggregates, were strongly stained with both C219 and MRK16 MoAb similar to the findings in tumour specimens. In one case of sarcoidosis and one case of lymphadenitis, CD68 antibodies were used on serial sections to confirm the macrophage nature of the P-gp expressing cells.

**Figure 1** High power magnification of a case of lymphocytic lymphoma (case no 15, Table III) showing many macrophages strongly positive for MRK16 monoclonal antibody. Note that lymphoma cells are clearly negative (ModAMex method with APAAP immunostaining × 44).

**Figure 2** Serial sections of malignant lymphoma (case no 9, Table III) with high content of macrophages. Area identified by a blood vessel (arrow). A – Staining of macrophages, some with vacuolated cytoplasm, by anti-P-gp, MRK16 antibody. No staining of lymphoid cells. B – Staining of a larger number of macrophages by Y2/131 (CD68), anti-macrophage antibody. However, four stained cells identical to those in A are shown with arrowheads (ModAMeX method with APAAP immunostaining × 40).

### Discussion

C219 and MRK16 have been used previously for characterisation of P-gp expression in normal tissues on cryostat sections (Thiebaut *et al.*, 1987; Sugawara *et al.*, 1988; Thiebaut *et al.*, 1989). Localisation of P-gp has been found on biliary canalicular front of hepatocytes, apical portions of proximal tubular cells of the kidney and diffusely in adrenal cortical and medullary cells with the two antibodies (Thiebaut *et al.*, 1987; Thiebaut *et al.*, 1989). On both cryostat sections and ModAMeX processed sections we found P-gp expression in normal tissues similar to that reported by these authors. P-gp expression by neoplastic cells in cases of renal-cell carcinoma, liver-cell carcinoma and ovarian carcinoma was expected from the reported Northern blot studies (Fojo *et al.*, 1987*b*; Goldstein, *et al.*, 1989; Moscow *et al.*, 1989). In addition P-gp expression could be identified on resistant cell lines compared to its absence on parental cell lines. These observations confirmed the reliability of immunohistochemical methods for studying P-gp expression in human cancers. Moreover, the strong reactivities observed in paraplast preparations showed that the ModAMeX technique, which was essentially developed for optimal preservation of leucocyte differentiation antigens, is equally feasible for the immunodetection of P-gp.

Patients with non-Hodgkin's lymphoma were selected on the basis of their resistance to drugs normally involved in the MDR phenotype. We were surprised to find the lack of P-gp expression by lymphoma cells in all cases. Our results are in conflict with those reported by Salmon *et al.* (1989) and Dalton *et al.* (1989), who found at least 50% positive cases (3/6 and 1/1 respectively) with a biotin-avidin conjugated immunoperoxidase method using JSB-1 and C219 MoAb. Such a discrepancy cannot be explained either by the selection of patients, or by the immunostaining methods, since, in our experience, the APAAP technique used in our study is as sensitive as the biotin-avidin procedure. It is possible that the heterogeneity of the results is due to the small size of the patient populations in previous studies. In a recent report, Sugawara *et al.* (1989) did not find significant staining of lymphoma cells by MRK16 MoAb using flow cytometry. Furthermore, Northern blot studies have shown that the incidence of MDR in lymphoma patients is rather low, accounting for only about 30% of the patients with moderate or low MDR1 RNA levels (Goldstein *et al.*, 1989; Moscow *et al.*, 1989). The latter findings are in line with our study and suggest that drug resistance to CHOP or CHOP-derived regimens in lymphomas could be mediated by mechanisms other than P-gp over expression.

P-gp expression in Hodgkin's disease has not been previously reported. In our study, Reed-Sternberg cells did not express P-gp. However, these patients had not been selected on the basis of their chemotherapeutic unresponsiveness, since 8 of 10 patients achieved complete remission after first line MOPP/ABVD treatment. Further studies on MOPP/ABVD resistant patients are needed to assess the possible role of MDR in refractory Hodgkin's disease.

Interestingly, we found positivity of numerous stromal cells in some non-lymphoid and lymphoid tumours (Figure 1) as well as in non-tumoural inflammatory processes. In these cases, macrophages, mainly in aggregates, were positive with both C219 and MRK16 MoAb. The reactivity appears specific because the two antibodies recognise two different membrane epitopes. The findings need to be strengthened further by *in situ* hybridisation with a specific MDR-1 probe (currently in progress). P-gp was not expressed by macrophages in all tumours, neither did all macrophages in the same tumour express P-gp. It is possible, therefore, that the positivity is due to the expression of P-gp by a subset of macrophages. In this context, cellular activation signals involved in antitumour host responses or inflammatory processes may trigger cells of the monocyte/macrophage system to induce P-gp expression. The mechanism of P-gp expression by macrophages remains to be elucidated but our findings suggest that these cells are responsible in some instances for an overestimation of the MDR content of tumour cells with Northern blot or immunoblot techniques performed on fresh tissues homogenates.

Finally, this study confirms that immunohistochemistry represents a useful tool for studying P-gp expression in human cancers, especially if the technique used allows a precise morphological assessment of antigen distribution and bypasses the interpretative difficulties of cryostat sections. However, immunomorphology does not allow exact quantification of P-gp content. Therefore, it may be advisable to combine immunohistochemical and molecular biology techniques for exploring any putative correlation between MDR expression and the clinical outcome.

This work was supported by grants from the Association pour la Recherche sur le Cancer (No 6229). The authors would like to thank Pr. J. Pris and Drs F. Huguet, M. Attal, C. Nouvel and H. Rubie for their cooperation and. M. Frede for his excellent secretarial assistance. We are also grateful to the technologists of the anatomical pathology laboratory for their patient collaboration and work.

### References

ANONYMOUS (1982). National Cancer Institute sponsored study of classifications of non-Hodgkin's lymphomas. Summary and description of a Working Formulation for clinical usage. *Cancer*, **49**, 2112.

BELL, D.R., GERLACH, J.H., KARTNER, N., BUICK, R.N. & LING, V. (1985). Detection of P-glycoprotein in ovarian cancer: a molecular marker associated with multidrug resistance. *J. Clin. Oncol.*, **3**, 311.

BRADLEY, G., JURANKA, P.F. & LING, V. (1988). Mechanism of multidrug resistance. *Biochim. Biophys. Acta*, **948**, 87.

CORDELL, J.L., FALINI, B., ERBER, W.N. & 6 others (1984). Immunoenzymatic labelling of monoclonal antibodies using immune complexes of alkaline phosphatase and monoclonal anti-alkaline phosphatase (APAAP complexes). *J. Histochem. Cytochem.*, **32**, 219.

DALTON, W.S., GROGAN, T.M., MELTZER, P.S. & 5 others (1989). Drug-resistance in multiple myeloma and non-Hodgkin's lymphoma: detection of P-glycoprotein and potential circumvention by addition of verapamil to chemotherapy. *J. Clin. Oncol.*, **7**, 415.

DELSOL, G., CHITTAL, S., BROUSSET, P. & 7 others (1989). Immunohistochemical demonstration of leucocyte differentiation antigens on paraffin sections using a modified AMeX (ModAMeX) method. *Histopathology*, **15**, 461.

FOJO, A.T., UEDA, K., SLAMON, D.J., POPLACK, D.G., GOTTESMAN, M.M. & PASTAN, I. (1987a). Expression of a multidrug-resistance gene in human tumors and tissues. *Proc. Natl Acad. Sci. USA*, **84**, 265.

FOJO, A.T., SHEN, D.W., MICKLEY, L.A., PASTAN, I. & GOTTESMAN, M.M. (1987b). Intrinsic drug resistance in human kidney cancer is associated with expression of a human multidrug-resistance gene. *J. Clin. Oncol.*, **5**, 1922.

GERLACH, J.H., BELL, D.R., KARAKOUSIS, C. & 5 others (1987). P-glycoprotein in human sarcoma: evidence for multidrug resistance. *J. Clin. Oncol.*, **5**, 1452.

GOLDSTEIN, L.J., GALSKI, H., FOJO, A. & 11 others (1989). Expression of a multidrug resistance gene in human cancers. *J. Natl Cancer Inst.*, **81**, 116.

HAMADA, H. & TSURUO, T. (1986). Functional role for the 170-to 180-kDa glycoprotein specific to drug-resistant tumor cells as revealed by monoclonal antibodies. *Proc. Natl Acad. Sci. USA*, **83**, 7785.

KARTNER, N., EVERNDEN-PORELLE, D., BRADLEY, G. & LING, V. (1985). Detection of P-glycoprotein in multidrug-resistant cell lines by monoclonal antibodies. *Nature*, **316**, 820.

KAYE, S.B. (1988). The multidrug resistance phenotype. *Br. J. Cancer*, **58**, 691.

LAURENT, G., ALSAATI, T., OLIVE, D., LAURENT, J.C., PONCELET, P. & DELSOL, G. (1986). Expression of Tac antigen in B-cell lymphomas. *Clin. Exp. Immunol.*, **65**, 354.

MARIE, J.P., BROPHY, N.A., BERRY, J.M. & 4 others (1989). Expression of the multidrug resistance gene mdr 1 in human leukemia and lymphoma cells: comparison of RNA slot blotting, *in situ* RNA hybridization, and detection of P-glycoprotein by immunocytochemistry. *Proc. Am. Assoc. Cancer Res.*, **30**, 497.

MOSCOW, J.A., FAIRCHILD, C.R., MADDEN, M.J. & 7 others (1989). Expression of anionic glutathione-S-transferase and P-glycoprotein genes in human tissues and tumors. *Cancer Res.*, **49**, 1422.

PASTAN, I. & GOTTESMAN, M. (1987). Multiple-drug resistance in human cancer. *N. Engl. J. Med.*, **316**, 1388.

SALMON, S.E., GROGAN, T.M., MILLER, T., SCHEPER, R. & DALTON, W.S. (1989). Prediction of doxorubicin resistance *in vitro* in myeloma, lymphoma, and breast cancer by P-glycoprotein staining. *J. Natl Cancer Inst.*, **81**, 696.

SATO, Y., MUKAI, K., WATANABE, S., GOTO, M. & SHIMOSATO, Y. (1986). The AMeX method. A simplified technique of tissue processing and paraffin embedding with improved preservation of antigens for immunostaining. *Am. J. Pathol.*, **125**, 431.

SCHEPER, R.J., BULTE, J.W.M., BRAKKEE, J.G.P. & 8 others (1988). Monoclonal antibody JSB-1 detects a highly conserved epitope on the P-glycoprotein associated with multi-drug-resistance. *Int. J. Cancer*, **42**, 389.

SHEN, D.W., PASTAN, I. & GOTTESMAN, M.M. (1988). *In situ* hybridization analysis of acquisition and loss of the human multidrug-resistance gene. *Cancer Res.*, **48**, 4334.

SUGAWARA, I., KATAOKA, I., MORISHITA, Y. & 4 others (1988). Tissue distribution of P-glycoprotein encoded by a multidrug-resistant gene as revealed by a monoclonal antibody, MRK 16. *Cancer Res.*, **48**, 1926.

SUGAWARA, I., KODO, H., OHKOCHI, E., HAMADA, H., TSURUO, T. & MORI, S. (1989). High-level expression of MRK16 and MRK20 murine monoclonal antibody-defined proteins (170,000–180,000 P-glycoprotein and 85,000 protein) in leukaemias and malignant lymphomas. *Br. J. Cancer*, **60**, 538.

THIEBAUT, F., TSURUO, T., HAMADA, H., GOTTESMAN, M.M., PASTAN, I. & WILLINGHAM, M.C. (1987). Cellular localization of the multidrug-resistance gene product P-glycoprotein in normal human tissues. *Proc. Natl Acad. Sci USA*, **84**, 7735.

THIEBAUT, F., TSURUO, T., HAMADA, H., GOTTESMAN, M.M. PASTAN, I. & WILLINGHAM, M.C. (1989). Immunohistochemical localization in normal tissues of different epitopes in the multidrug transport protein P 170: evidence for localization in brain capillaries and crossreactivity of one antibody with a muscle protein. *J. Histochem. Cytochem.*, **37**, 159.


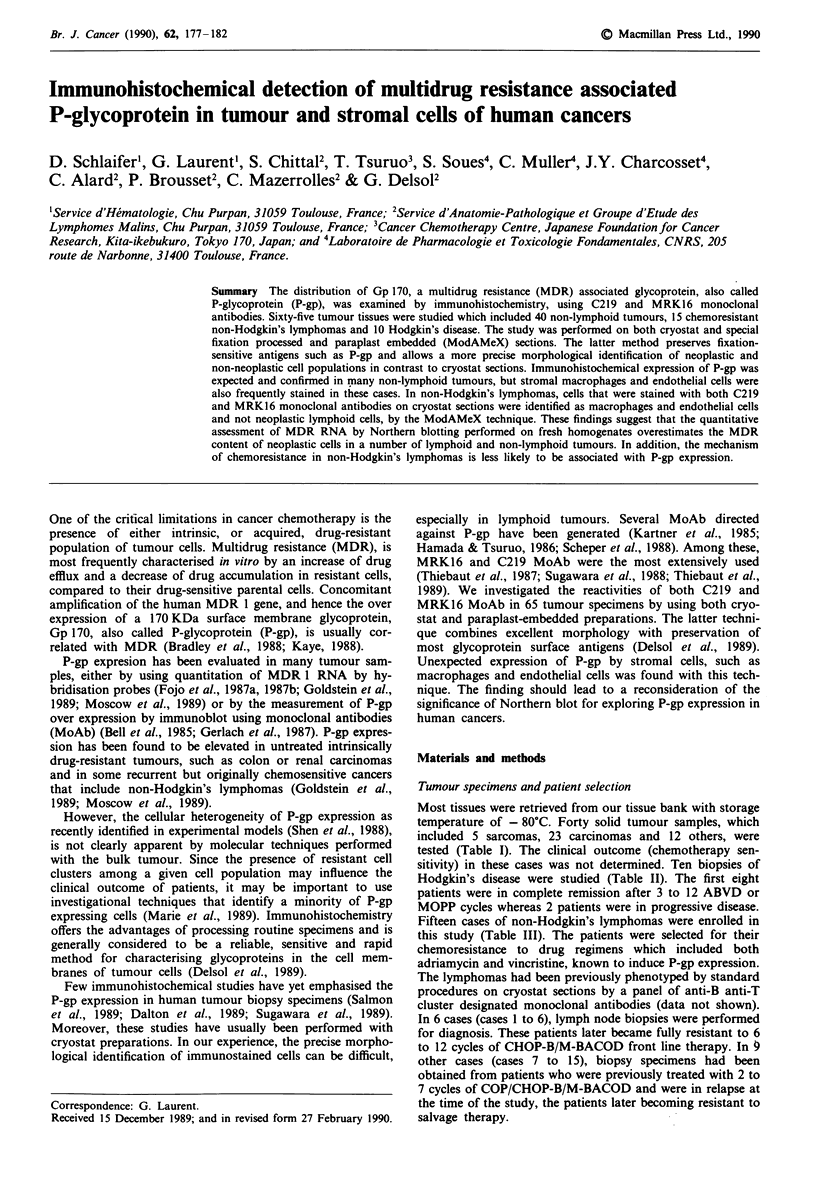

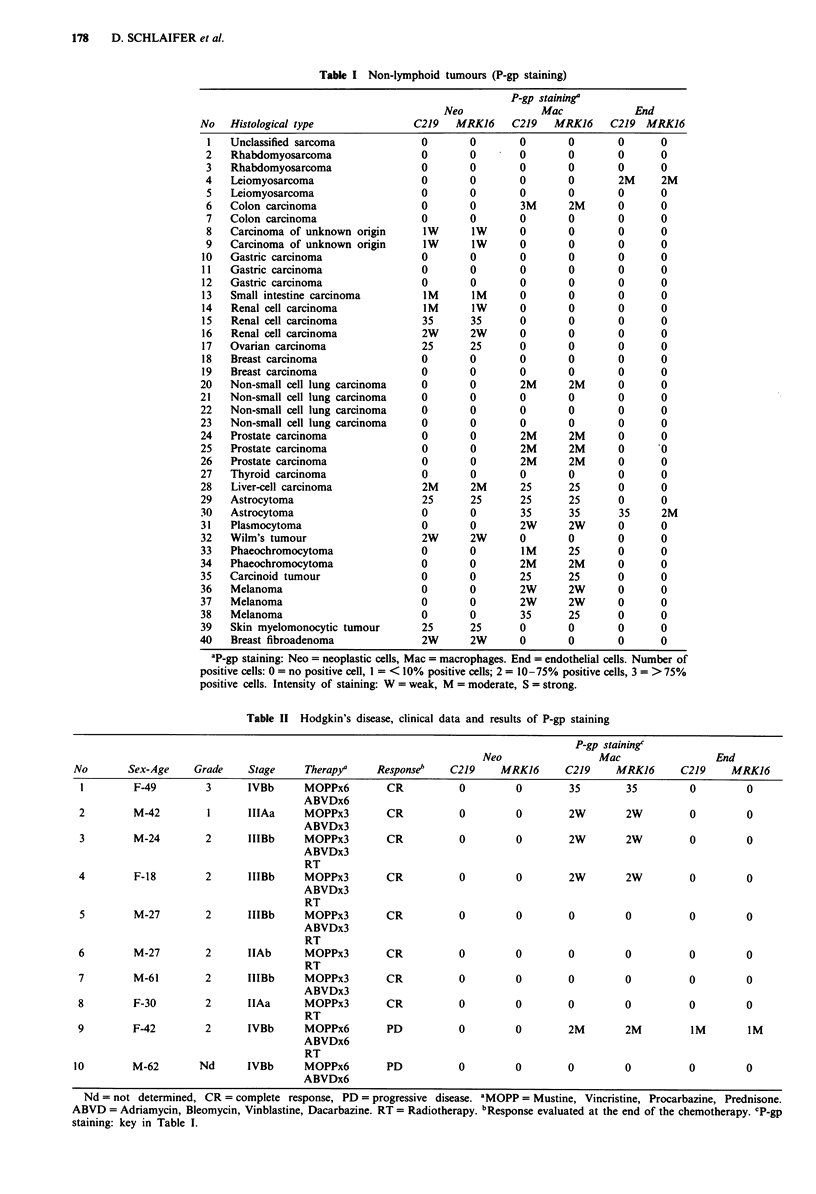

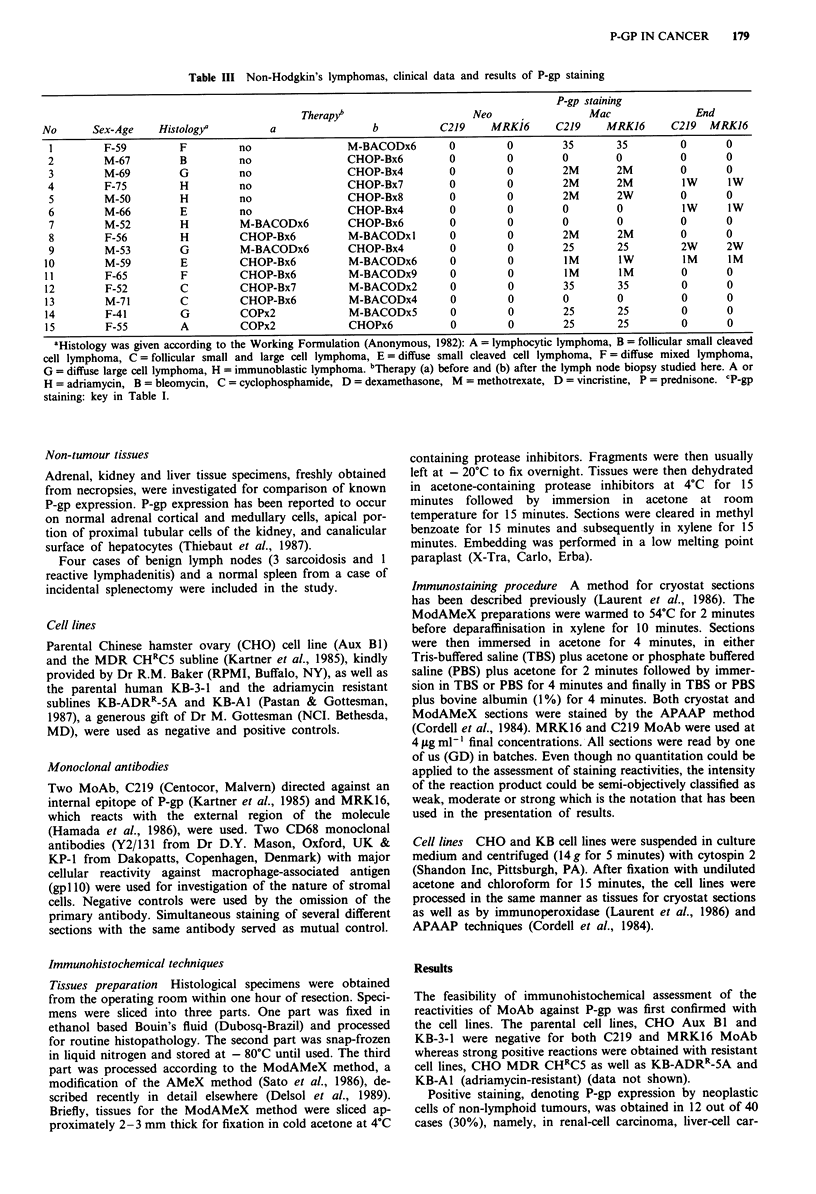

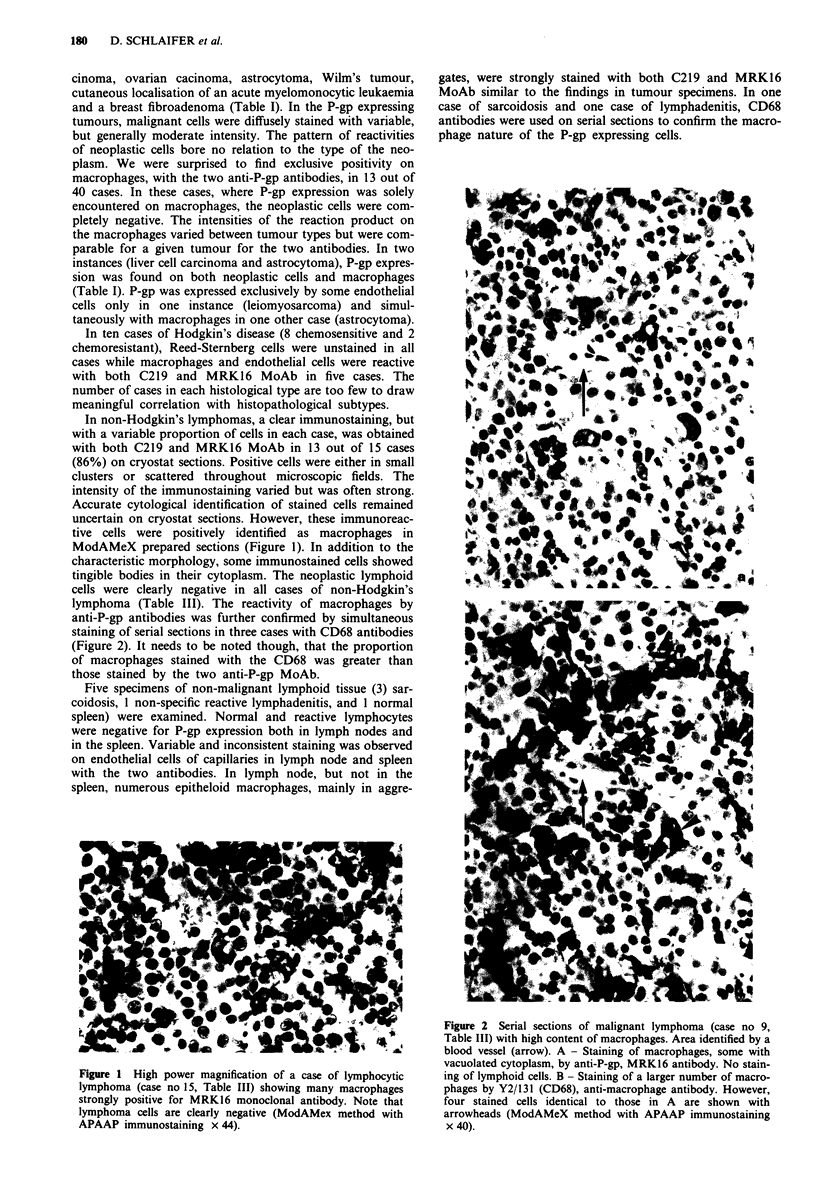

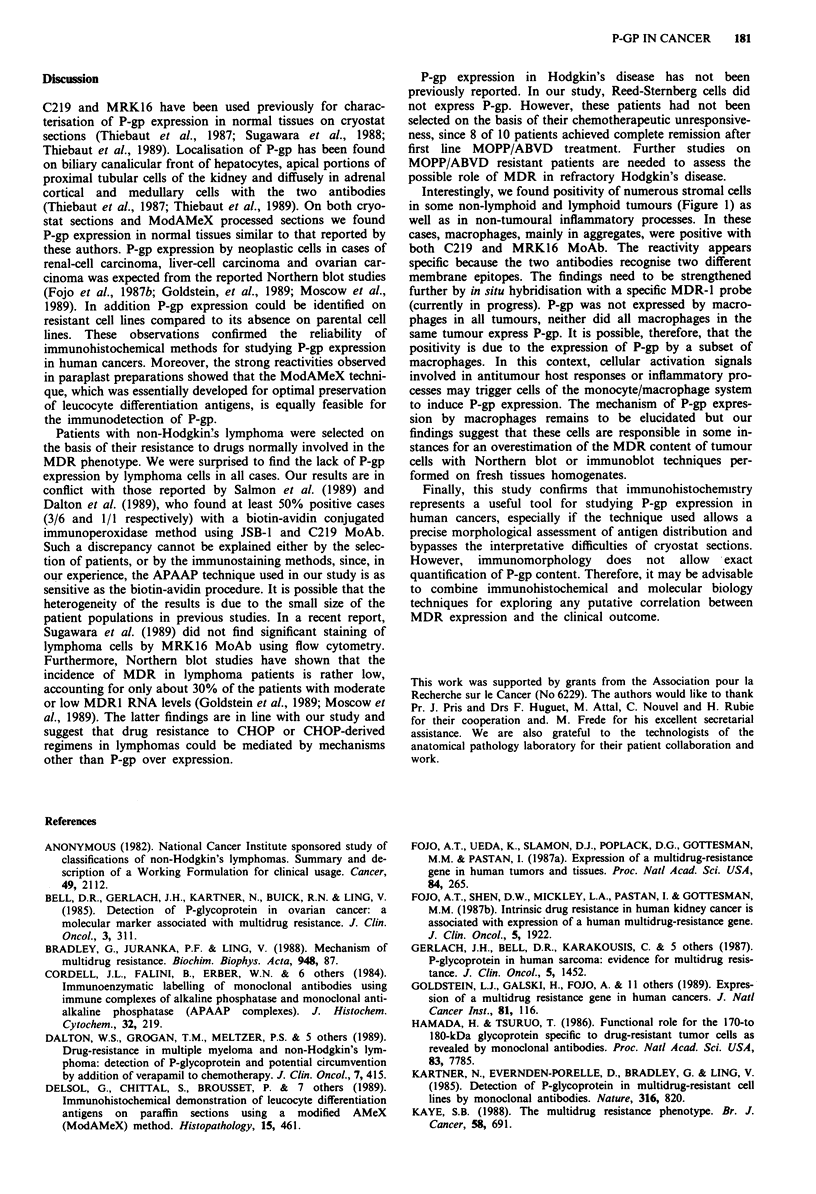

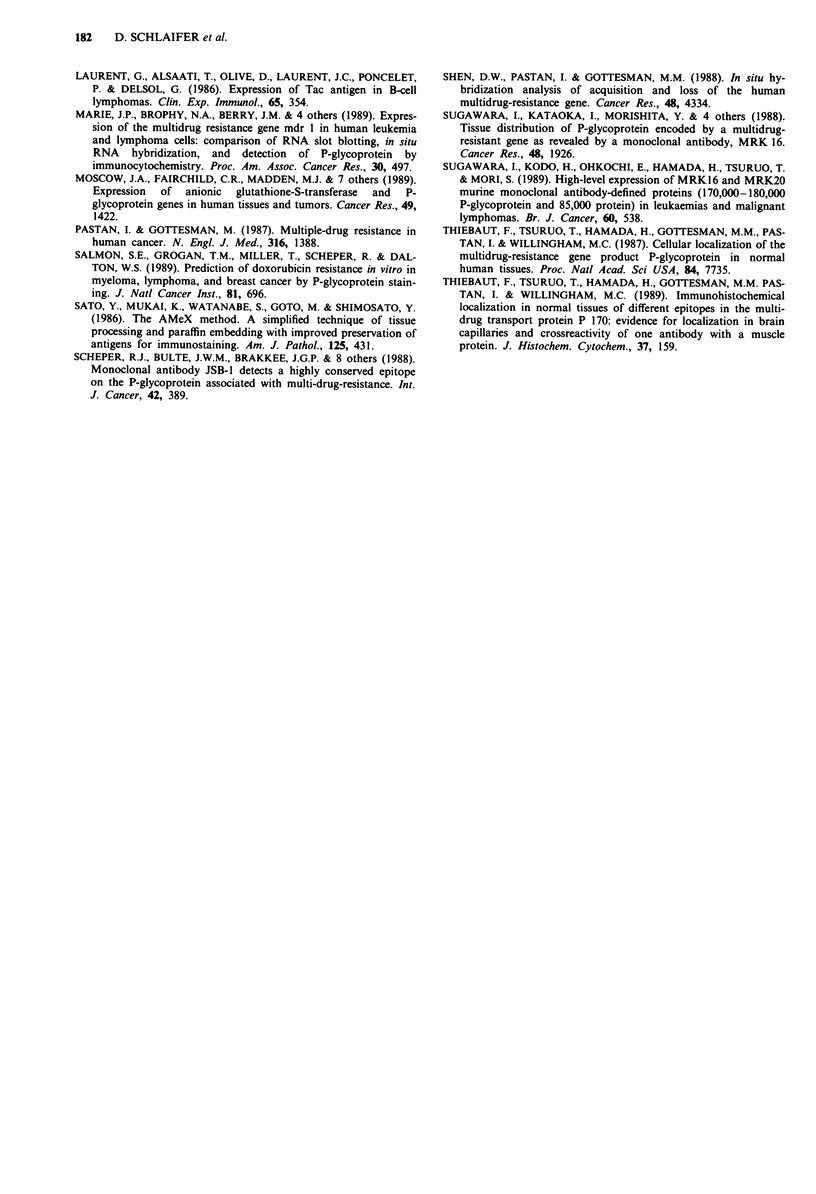


## References

[OCR_00565] Bell D. R., Gerlach J. H., Kartner N., Buick R. N., Ling V. (1985). Detection of P-glycoprotein in ovarian cancer: a molecular marker associated with multidrug resistance.. J Clin Oncol.

[OCR_00571] Bradley G., Juranka P. F., Ling V. (1988). Mechanism of multidrug resistance.. Biochim Biophys Acta.

[OCR_00577] Cordell J. L., Falini B., Erber W. N., Ghosh A. K., Abdulaziz Z., MacDonald S., Pulford K. A., Stein H., Mason D. Y. (1984). Immunoenzymatic labeling of monoclonal antibodies using immune complexes of alkaline phosphatase and monoclonal anti-alkaline phosphatase (APAAP complexes).. J Histochem Cytochem.

[OCR_00582] Dalton W. S., Grogan T. M., Meltzer P. S., Scheper R. J., Durie B. G., Taylor C. W., Miller T. P., Salmon S. E. (1989). Drug-resistance in multiple myeloma and non-Hodgkin's lymphoma: detection of P-glycoprotein and potential circumvention by addition of verapamil to chemotherapy.. J Clin Oncol.

[OCR_00587] Delsol G., Chittal S., Brousset P., Caveriviere P., Roda D., Mazerolles C., Barillet-Alard C., al Saati T., Gorguet B., Voigt J. J. (1989). Immunohistochemical demonstration of leucocyte differentiation antigens on paraffin sections using a modified AMeX (ModAMeX) method.. Histopathology.

[OCR_00599] Fojo A. T., Shen D. W., Mickley L. A., Pastan I., Gottesman M. M. (1987). Intrinsic drug resistance in human kidney cancer is associated with expression of a human multidrug-resistance gene.. J Clin Oncol.

[OCR_00593] Fojo A. T., Ueda K., Slamon D. J., Poplack D. G., Gottesman M. M., Pastan I. (1987). Expression of a multidrug-resistance gene in human tumors and tissues.. Proc Natl Acad Sci U S A.

[OCR_00605] Gerlach J. H., Bell D. R., Karakousis C., Slocum H. K., Kartner N., Rustum Y. M., Ling V., Baker R. M. (1987). P-glycoprotein in human sarcoma: evidence for multidrug resistance.. J Clin Oncol.

[OCR_00610] Goldstein L. J., Galski H., Fojo A., Willingham M., Lai S. L., Gazdar A., Pirker R., Green A., Crist W., Brodeur G. M. (1989). Expression of a multidrug resistance gene in human cancers.. J Natl Cancer Inst.

[OCR_00615] Hamada H., Tsuruo T. (1986). Functional role for the 170- to 180-kDa glycoprotein specific to drug-resistant tumor cells as revealed by monoclonal antibodies.. Proc Natl Acad Sci U S A.

[OCR_00621] Kartner N., Evernden-Porelle D., Bradley G., Ling V. Detection of P-glycoprotein in multidrug-resistant cell lines by monoclonal antibodies.. Nature.

[OCR_00626] Kaye S. B. (1988). The multidrug resistance phenotype.. Br J Cancer.

[OCR_00632] Laurent G., Al Saati T., Olive D., Laurent J. C., Poncelet P., Delsol G. (1986). Expression of Tac antigen in B cell lymphomas.. Clin Exp Immunol.

[OCR_00644] Moscow J. A., Fairchild C. R., Madden M. J., Ransom D. T., Wieand H. S., O'Brien E. E., Poplack D. G., Cossman J., Myers C. E., Cowan K. H. (1989). Expression of anionic glutathione-S-transferase and P-glycoprotein genes in human tissues and tumors.. Cancer Res.

[OCR_00650] Pastan I., Gottesman M. (1987). Multiple-drug resistance in human cancer.. N Engl J Med.

[OCR_00656] Salmon S. E., Grogan T. M., Miller T., Scheper R., Dalton W. S. (1989). Prediction of doxorubicin resistance in vitro in myeloma, lymphoma, and breast cancer by P-glycoprotein staining.. J Natl Cancer Inst.

[OCR_00660] Sato Y., Mukai K., Watanabe S., Goto M., Shimosato Y. (1986). The AMeX method. A simplified technique of tissue processing and paraffin embedding with improved preservation of antigens for immunostaining.. Am J Pathol.

[OCR_00666] Scheper R. J., Bulte J. W., Brakkee J. G., Quak J. J., van der Schoot E., Balm A. J., Meijer C. J., Broxterman H. J., Kuiper C. M., Lankelma J. (1988). Monoclonal antibody JSB-1 detects a highly conserved epitope on the P-glycoprotein associated with multi-drug-resistance.. Int J Cancer.

[OCR_00672] Shen D. W., Pastan I., Gottesman M. M. (1988). In situ hybridization analysis of acquisition and loss of the human multidrug-resistance gene.. Cancer Res.

[OCR_00677] Sugawara I., Kataoka I., Morishita Y., Hamada H., Tsuruo T., Itoyama S., Mori S. (1988). Tissue distribution of P-glycoprotein encoded by a multidrug-resistant gene as revealed by a monoclonal antibody, MRK 16.. Cancer Res.

[OCR_00683] Sugawara I., Kodo H., Ohkochi E., Hamada H., Tsuruo T., Mori S. (1989). High-level expression of MRK 16 and MRK 20 murine monoclonal antibody-define proteins (170,000-180,000 P-glycoprotein and 85,000 protein) in leukaemias and malignant lymphomas.. Br J Cancer.

[OCR_00692] Thiebaut F., Tsuruo T., Hamada H., Gottesman M. M., Pastan I., Willingham M. C. (1987). Cellular localization of the multidrug-resistance gene product P-glycoprotein in normal human tissues.. Proc Natl Acad Sci U S A.

[OCR_00698] Thiebaut F., Tsuruo T., Hamada H., Gottesman M. M., Pastan I., Willingham M. C. (1989). Immunohistochemical localization in normal tissues of different epitopes in the multidrug transport protein P170: evidence for localization in brain capillaries and crossreactivity of one antibody with a muscle protein.. J Histochem Cytochem.

