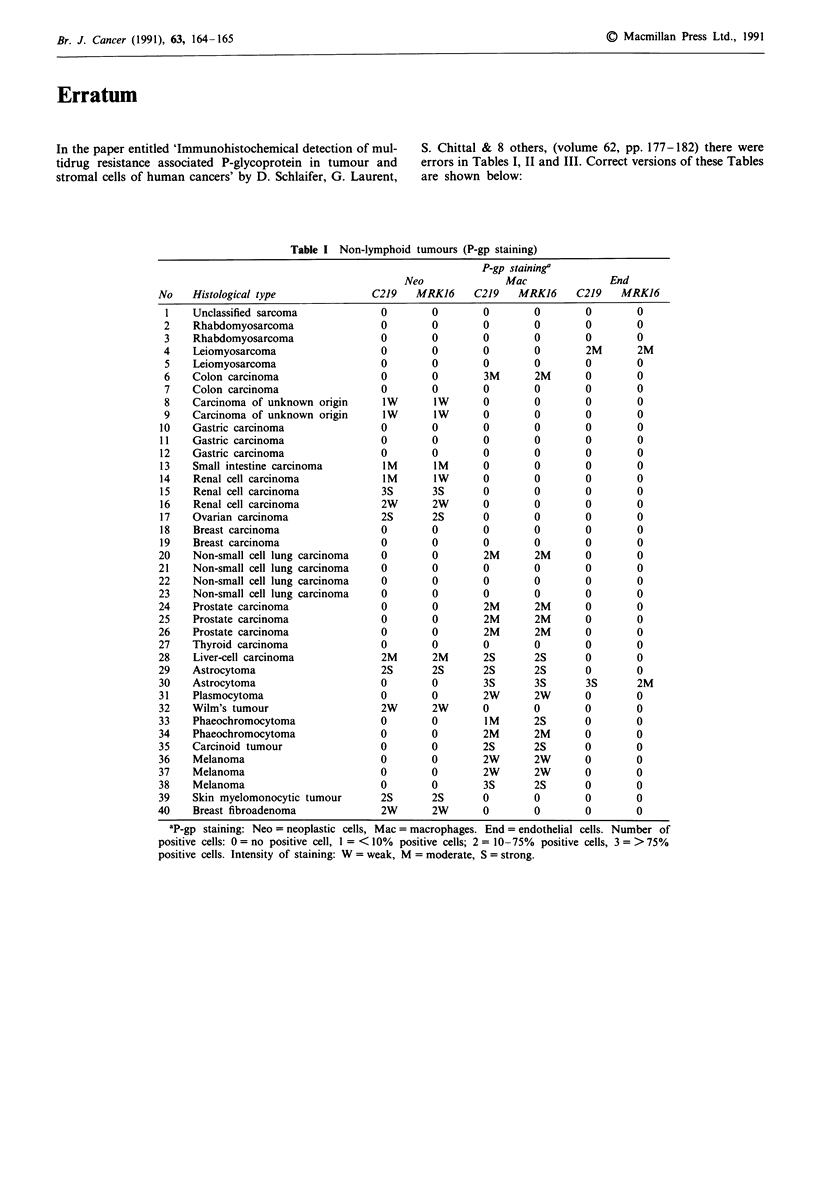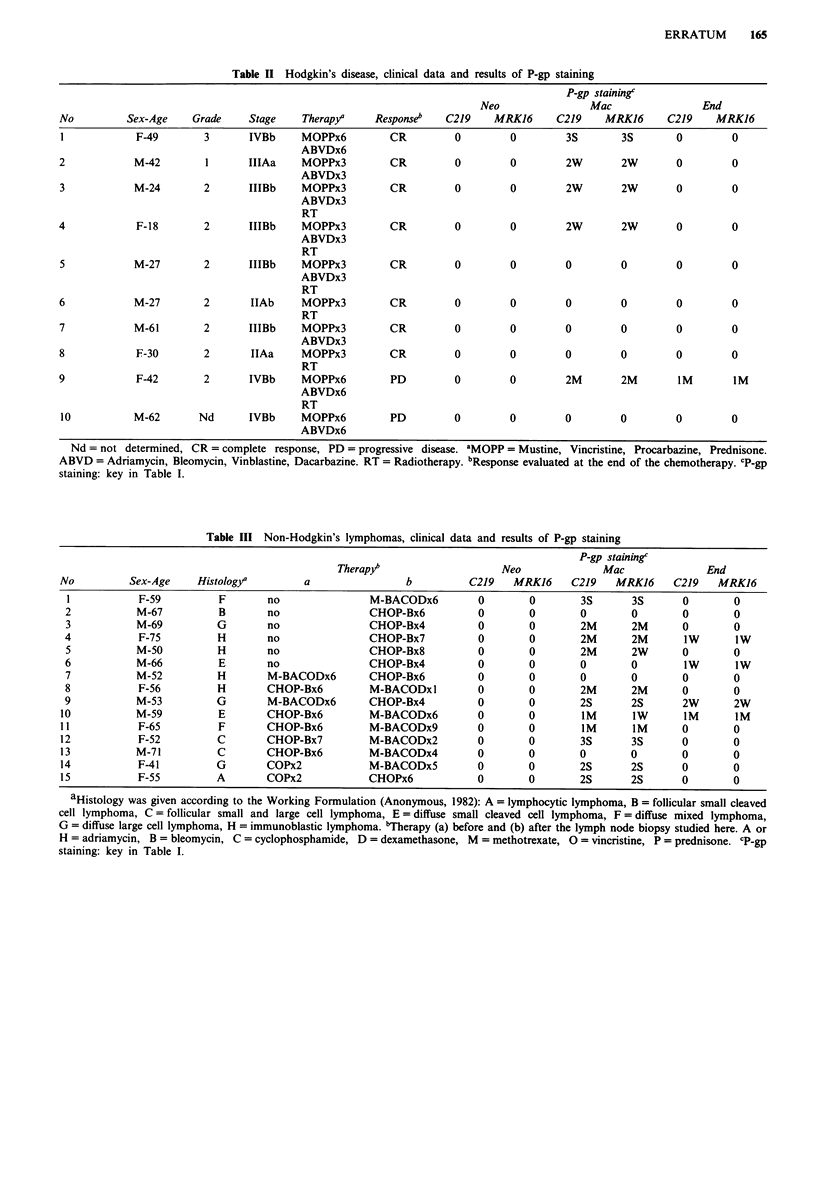# Erratum

**Published:** 1991-01

**Authors:** 


Br. J. Cancer (1991), 63, 164–165 © Macmillan Press Ltd., 1991

### Erratum

In the paper entitled 'Immunohistochemical detection of multidrug resistance associated P-glycoprotein in tumour and stromal cells of human cancers' by D. Schlaifer, G. Laurent, S. Chittal & 8 others, (volume 62, pp. 177–182) there were errors in Tables I, II and III. Correct versions of these Tables are shown below:

**Table I** Non-lymphoid tumours (P-gp staining)

| | | P-gp staining[a] | | | | | |
|---|---|---|---|---|---|---|---|
| | | Neo | | Mac | | End | |
| No | Histological type | C219 | MRK16 | C219 | MRK16 | C219 | MRK16 |
| 1 | Unclassified sarcoma | 0 | 0 | 0 | 0 | 0 | 0 |
| 2 | Rhabdomyosarcoma | 0 | 0 | 0 | 0 | 0 | 0 |
| 3 | Rhabdomyosarcoma | 0 | 0 | 0 | 0 | 0 | 0 |
| 4 | Leiomyosarcoma | 0 | 0 | 0 | 0 | 2M | 2M |
| 5 | Leiomyosarcoma | 0 | 0 | 0 | 0 | 0 | 0 |
| 6 | Colon carcinoma | 0 | 0 | 3M | 2M | 0 | 0 |
| 7 | Colon carcinoma | 0 | 0 | 0 | 0 | 0 | 0 |
| 8 | Carcinoma of unknown origin | 1W | 1W | 0 | 0 | 0 | 0 |
| 9 | Carcinoma of unknown origin | 1W | 1W | 0 | 0 | 0 | 0 |
| 10 | Gastric carcinoma | 0 | 0 | 0 | 0 | 0 | 0 |
| 11 | Gastric carcinoma | 0 | 0 | 0 | 0 | 0 | 0 |
| 12 | Gastric carcinoma | 0 | 0 | 0 | 0 | 0 | 0 |
| 13 | Small intestine carcinoma | 1M | 1M | 0 | 0 | 0 | 0 |
| 14 | Renal cell carcinoma | 1M | 1W | 0 | 0 | 0 | 0 |
| 15 | Renal cell carcinoma | 3S | 3S | 0 | 0 | 0 | 0 |
| 16 | Renal cell carcinoma | 2W | 2W | 0 | 0 | 0 | 0 |
| 17 | Ovarian carcinoma | 2S | 2S | 0 | 0 | 0 | 0 |
| 18 | Breast carcinoma | 0 | 0 | 0 | 0 | 0 | 0 |
| 19 | Breast carcinoma | 0 | 0 | 0 | 0 | 0 | 0 |
| 20 | Non-small cell lung carcinoma | 0 | 0 | 2M | 2M | 0 | 0 |
| 21 | Non-small cell lung carcinoma | 0 | 0 | 0 | 0 | 0 | 0 |
| 22 | Non-small cell lung carcinoma | 0 | 0 | 0 | 0 | 0 | 0 |
| 23 | Non-small cell lung carcinoma | 0 | 0 | 0 | 0 | 0 | 0 |
| 24 | Prostate carcinoma | 0 | 0 | 2M | 2M | 0 | 0 |
| 25 | Prostate carcinoma | 0 | 0 | 2M | 2M | 0 | 0 |
| 26 | Prostate carcinoma | 0 | 0 | 2M | 2M | 0 | 0 |
| 27 | Thyroid carcinoma | 0 | 0 | 0 | 0 | 0 | 0 |
| 28 | Liver-cell carcinoma | 2M | 2M | 2S | 2S | 0 | 0 |
| 29 | Astrocytoma | 2S | 2S | 2S | 2S | 0 | 0 |
| 30 | Astrocytoma | 0 | 0 | 3S | 3S | 3S | 2M |
| 31 | Plasmocytoma | 0 | 0 | 2W | 2W | 0 | 0 |
| 32 | Wilm's tumour | 2W | 2W | 0 | 0 | 0 | 0 |
| 33 | Phaeochromocytoma | 0 | 0 | 1M | 2S | 0 | 0 |
| 34 | Phaeochromocytoma | 0 | 0 | 2M | 2M | 0 | 0 |
| 35 | Carcinoid tumour | 0 | 0 | 2S | 2S | 0 | 0 |
| 36 | Melanoma | 0 | 0 | 2W | 2W | 0 | 0 |
| 37 | Melanoma | 0 | 0 | 2W | 2W | 0 | 0 |
| 38 | Melanoma | 0 | 0 | 3S | 2S | 0 | 0 |
| 39 | Skin myelomonocytic tumour | 2S | 2S | 0 | 0 | 0 | 0 |
| 40 | Breast fibroadenoma | 2W | 2W | 0 | 0 | 0 | 0 |

[a]P-gp staining: Neo = neoplastic cells, Mac = macrophages. End = endothelial cells. Number of positive cells: 0 = no positive cell, 1 = < 10% positive cells; 2 = 10–75% positive cells, 3 = > 75% positive cells. Intensity of staining: W = weak, M = moderate, S = strong.

**Table II** Hodgkin's disease, clinical data and results of P-gp staining

| No | Sex-Age | Grade | Stage | Therapy[a] | Response[b] | P-gp staining[c] Neo C219 | Neo MRK16 | Mac C219 | Mac MRK16 | End C219 | End MRK16 |
|---|---|---|---|---|---|---|---|---|---|---|---|
| 1 | F-49 | 3 | IVBb | MOPPx6 ABVDx6 | CR | 0 | 0 | 3S | 3S | 0 | 0 |
| 2 | M-42 | 1 | IIIAa | MOPPx3 ABVDx3 | CR | 0 | 0 | 2W | 2W | 0 | 0 |
| 3 | M-24 | 2 | IIIBb | MOPPx3 ABVDx3 RT | CR | 0 | 0 | 2W | 2W | 0 | 0 |
| 4 | F-18 | 2 | IIIBb | MOPPx3 ABVDx3 RT | CR | 0 | 0 | 2W | 2W | 0 | 0 |
| 5 | M-27 | 2 | IIIBb | MOPPx3 ABVDx3 RT | CR | 0 | 0 | 0 | 0 | 0 | 0 |
| 6 | M-27 | 2 | IIAb | MOPPx3 RT | CR | 0 | 0 | 0 | 0 | 0 | 0 |
| 7 | M-61 | 2 | IIIBb | MOPPx3 ABVDx3 | CR | 0 | 0 | 0 | 0 | 0 | 0 |
| 8 | F-30 | 2 | IIAa | MOPPx3 RT | CR | 0 | 0 | 0 | 0 | 0 | 0 |
| 9 | F-42 | 2 | IVBb | MOPPx6 ABVDx6 RT | PD | 0 | 0 | 2M | 2M | 1M | 1M |
| 10 | M-62 | Nd | IVBb | MOPPx6 ABVDx6 | PD | 0 | 0 | 0 | 0 | 0 | 0 |

Nd = not determined, CR = complete response, PD = progressive disease. [a]MOPP = Mustine, Vincristine, Procarbazine, Prednisone. ABVD = Adriamycin, Bleomycin, Vinblastine, Dacarbazine. RT = Radiotherapy. [b]Response evaluated at the end of the chemotherapy. [c]P-gp staining: key in Table I.

**Table III** Non-Hodgkin's lymphomas, clinical data and results of P-gp staining

| No | Sex-Age | Histology[a] | Therapy[b] a | Therapy[b] b | P-gp staining[c] Neo C219 | Neo MRK16 | Mac C219 | Mac MRK16 | End C219 | End MRK16 |
|---|---|---|---|---|---|---|---|---|---|---|
| 1 | F-59 | F | no | M-BACODx6 | 0 | 0 | 3S | 3S | 0 | 0 |
| 2 | M-67 | B | no | CHOP-Bx6 | 0 | 0 | 0 | 0 | 0 | 0 |
| 3 | M-69 | G | no | CHOP-Bx4 | 0 | 0 | 2M | 2M | 0 | 0 |
| 4 | F-75 | H | no | CHOP-Bx7 | 0 | 0 | 2M | 2M | 1W | 1W |
| 5 | M-50 | H | no | CHOP-Bx8 | 0 | 0 | 2M | 2W | 0 | 0 |
| 6 | M-66 | E | no | CHOP-Bx4 | 0 | 0 | 0 | 0 | 1W | 1W |
| 7 | M-52 | H | M-BACODx6 | CHOP-Bx6 | 0 | 0 | 0 | 0 | 0 | 0 |
| 8 | F-56 | H | CHOP-Bx6 | M-BACODx1 | 0 | 0 | 2M | 2M | 0 | 0 |
| 9 | M-53 | G | M-BACODx6 | CHOP-Bx4 | 0 | 0 | 2S | 2S | 2W | 2W |
| 10 | M-59 | E | CHOP-Bx6 | M-BACODx6 | 0 | 0 | 1M | 1W | 1M | 1M |
| 11 | F-65 | F | CHOP-Bx6 | M-BACODx9 | 0 | 0 | 1M | 1M | 0 | 0 |
| 12 | F-52 | C | CHOP-Bx7 | M-BACODx2 | 0 | 0 | 3S | 3S | 0 | 0 |
| 13 | M-71 | C | CHOP-Bx6 | M-BACODx4 | 0 | 0 | 0 | 0 | 0 | 0 |
| 14 | F-41 | G | COPx2 | M-BACODx5 | 0 | 0 | 2S | 2S | 0 | 0 |
| 15 | F-55 | A | COPx2 | CHOPx6 | 0 | 0 | 2S | 2S | 0 | 0 |

[a]Histology was given according to the Working Formulation (Anonymous, 1982): A = lymphocytic lymphoma, B = follicular small cleaved cell lymphoma, C = follicular small and large cell lymphoma, E = diffuse small cleaved cell lymphoma, F = diffuse mixed lymphoma, G = diffuse large cell lymphoma, H = immunoblastic lymphoma. [b]Therapy (a) before and (b) after the lymph node biopsy studied here. A or H = adriamycin, B = bleomycin, C = cyclophosphamide, D = dexamethasone, M = methotrexate, O = vincristine, P = prednisone. [c]P-gp staining: key in Table I.